# Is it safe enough? A pilot feasibility study of an 8-day intensive treatment for severe PTSD

**DOI:** 10.3389/fpsyt.2023.1200411

**Published:** 2023-07-20

**Authors:** Hannes Gahnfelt, Per F. G. Carlsson, Christina Blomdahl

**Affiliations:** ^1^Department of Research, Education and Innovation, Södra Älvsborg Hospital, Region Västra Götaland, Borås, Sweden; ^2^R&D Centre Södra Älvsborg, Research, Education, Development & Innovation, Region Västra Götaland, Borås, Sweden

**Keywords:** PTSD, intensive treatment, PE, EMDR, physical activity

## Abstract

Intensive treatments for posttraumatic stress disorder (PTSD) are gaining increased research support. Treatment models targeting Complex PTSD and previously treatment-resistant PTSD have shown a good effect. A pilot study was performed to assess the feasibility of an 8-day intensive treatment program for severe PTSD in a Swedish public healthcare setting. Eleven participants completed treatment, and overall, the reduction in PTSD symptoms was considerable. Also, loss of diagnosis at 3-month follow-up was 100%. No adverse events occurred, and no elevation of suicidal intentions was reported. Symptom exacerbation could not be observed in the data and dropout due to the intensity of the treatment format did not occur. Based on these positive results, it is recommended that further research with larger samples is conducted. If found safe and effective, the 8-day treatment program could be an important addition to psychiatric healthcare.

## 1. Introduction

Posttraumatic stress disorder (PTSD) is a psychiatric condition that may develop after exposure to a traumatic event (1). PTSD is characterized by intrusions, efforts to avoid reminders of the traumatic event, changes in cognition and mood, and hyperarousal. The effectiveness of psychological treatments for PTSD has been well documented, including RCT studies (2, 3). The International Society of Traumatic Stress Studies recommends several forms of therapy for PTSD (4), e.g., eye movement desensitization and reprocessing therapy (EMDR) (5) and prolonged exposure (PE) (6). More recently, research has suggested that an increased frequency of sessions could further enhance treatment effects (7). Various forms of intensive PTSD treatments where several sessions per week were scheduled rather than once a week, have shown promising results (8, 9). Recently, intensive treatment for PTSD has also shown maintained effects in long-term follow-up (10). An important task, however, is not only to develop more effective treatments for PTSD in general but also to seek ways to relieve more severe forms of PTSD. This challenge has been addressed by combining EMDR, PE, physical activity, and psychoeducation in an 8-day intensive treatment program (ITP) (11). Despite most participants having previous, non-successful treatments for PTSD and multiple comorbidities, results were encouraging. Complex PTSD (C-PTSD) is a condition typically associated with repeated or extremely stressful traumatic events and is characterized by severe problems with affect regulation, distorted self-image and difficulties sustaining relationships (12). This group of patients has also benefited from the 8-day ITP (13). Apart from significant symptom relief and more than 70% loss of PTSD and C-PTSD diagnosis, fears of problems and risks due to the intensive format were allayed: no suicide attempts or hospital admissions occurred. Also, no elevation of drop-outs due to the intensive format could be observed. Thus – intensifying PTSD treatment could also be useful for patients with more severe symptoms, including patients with comorbid borderline personality disorder (14). While the 8-day ITP is thoroughly evaluated in a clinic specialized in this specific treatment, questions remain about whether it can be implemented in different kinds of healthcare settings. A recent study in a Norwegian psychiatric clinic showed positive results in treating patients with PTSD with the 8-day ITP (15), but this specific treatment model has not yet been evaluated in any Swedish health care.

The aim of this pilot study was to examine whether the 8-day IPT is safe and feasible when applied in a Swedish psychiatric clinic for patients with either: C-PTSD, PTSD with previous unsuccessful treatment or PTSD with at least one comorbid psychiatric disorder. This population is labeled severe PTSD in this study. Furthermore, while no adverse events such as hospital admission and suicide attempts have been reported in previous studies, potential change in participants’ suicidal ideations during the 8-day ITP has not been monitored to our knowledge. Expanding knowledge on potential adverse effects on suicidal ideation during the 8-day IPT was also part of the research aim.

Specifically, we wanted to examine whether a change in symptoms of PTSD could be observed after the 8-day ITP. During the treatment course, PTSD symptoms and suicidal ideation were assessed as indicators of adverse treatment effects.

## 2. Method

The study was designed as an experimental open clinical study with no control group. Analyzes were conducted per protocol (16). The identity of the participants was blinded during data management procedures and data analysis.

### 2.1. Participants

All patients referred to the psychiatric outpatient clinic 15 at Södra Älvsborgs Sjukhus between August and December 2021 were assessed with a test version of the International Trauma Interview 3.2 (ITI) (17) for diagnosis of PTSD and C-PTSD. Inclusion criteria were: C-PTSD according to ICD-11, PTSD with at least one comorbid disorder requiring psychiatric care or PTSD with previous unsuccessful trauma-focused treatment. Exclusion criteria were: insufficient knowledge of the Swedish language to complete questionnaires, ongoing substance abuse, use of benzodiazepines, acute suicidality, or dissociative identity disorder (DID). No patients were excluded on these criteria during the inclusion phase. Of 29 eligible patients, 17 declined to participate in the 8-day ITP. 12 patients accepted the ITP. The final sample was 11 participants, as one participant dropped out on day 4 due to current stress and increased risk factors in the home environment during treatment. This participant did not attend posttreatment evaluation and was not included in the data analysis.

### 2.2. Ethics statement

All procedures were approved by the Ethics Committee in Lund on 2021-06-15. Informed consent was obtained before inclusion in the study. All patients who met the inclusion criteria obtained information about the study verbally and in writing. Accepting participation was not necessary to receive treatment.

### 2.3. Data collection

Screening for eligibility was the first step. Data collection started if the participant met the inclusion criteria, accepted to participate in the study and signed the informed consent (see Table 1). Between the last day of the treatment program and 3-month follow-up, no further interventions towards PTSD were initiated. Two participants did not complete measures of symptoms for multiple sessions (4 and 5, respectively) as they ended treatment prematurely due to reduced symptoms. The two participants completed posttreatment evaluation. Self-reported data were collected using REDCap (Research Electronic Data Capture) (18, 19), an electronic data capture tool hosted at Gothia Forum REDCap (RRID: SCR_003445). Participants entered symptom ratings *via* a QR code or web link. Participants who were uncomfortable using electronic devices were offered paper versions of self-report measures.

**Table 1:** Summary of assessment tools and time points.

Pretreatment	Day 1, 2, 3	Day 4	Day 5, 6, 7	Posttreatment	3-month follow-up
LEC-5 ITI SSI DES-II SCID-D^a^ PCL-5 ITQ-part 2	PCL-5	PCL-5 ITQ-part 2 SSI	PCL-5	ITI SSI PCL-5 ITQ-part 2	ITI SSI PCL-5 ITQ-part 2

a In case of elevated DES II score and suspected dissociative identity disorder.

LEC-5, Life Events Checklist; ITI, International Trauma Interview (test version 3.2); SSI, Beck’s Scale Suicide Ideation; DES-II, Dissociative Experience Scale II; SCID-D, Structured Clinical Interview for DSM-IV (R) Dissociative Disorders; PCL-5, Post Traumatic Checklist for DSM-5; ITQ-del 2, International Trauma Questionnaire part 2.

### 2.4. Measures

The primary outcome measure was the Post Traumatic Checklist for DSM-5 (PCL-5) (20), a self-report questionnaire for PTSD symptoms. PCL-5 has shown good internal reliability (0.96) and test–retest reliability (21), with good sensitivity to clinical change (22). The International Trauma Questionnaire (ITQ) (23) is a questionnaire in two parts developed to measure PTSD symptoms and Disturbance in Self Organization (DSO) symptoms (associated with C-PTSD). ITQ has shown high internal reliability (0.89 to 0.94) (24). Life Events Checklist for DSM-5 (LEC-5) (25) was used to identify traumatic events. LEC-5 has shown varying test–retest reliability between items (kappa = 0.36–0.80) (26).

To assess diagnostic criteria for PTSD and C-PTSD, a test version of the International Trauma Interview 3.2 (ITI) (17) was used. ITI has satisfying interrater reliability (α = 0.76) (27). Beck’s scale for suicide ideation (SSI) (28) in Swedish translation was used to screen high levels of suicidal intentions at pretreatment and to assess change in suicidal risk during the treatment. SSI has shown good internal reliability (0.89) (29). The Swedish translation of Dissociative Experience Scale II (DES) (30) was used to screen dissociative symptoms. DES II has good internal reliability (0.87) (30). Structured Clinical Interview for DSM-IV (R) Dissociative Disorders (SCID-D) (31) is a diagnostic interview for dissociative syndromes and was used to identify diagnosis of DID if scores on DES II indicated pathological dissociation. SCID-D has shown good interrater reliability (kappa = 0.72) and test–retest reliability (kappa = 0.88) (32).

### 2.5. Intervention

The 8-day ITP included PE, EMDR, physical activity and psychoeducation, in accordance with the treatment program developed at PSYTREC and described by Van Woudenberg et al. (11) During each day of treatment, the participant had 90 min of PE (6) in the morning and 90 min of EMDR (5) in the afternoon. Both treatments were slightly modified to adapt to the intensive format. *In vivo* exposure between sessions was removed from PE and stabilization phase prior to trauma processing in EMDR was removed. Prior to and after each PE and EMDR session, participants had 30–60 min of physical activity guided by a physiotherapist. The purpose of the physical activity was to help participants to increase body awareness, regulate physiological tension and counteract rumination and passive re-experiencing of trauma. The physical activities included high-pulse exercise and relaxation, e.g., boxing, medicine-ball exercise, tai-chi, mindful forest walk. In the evening, psychoeducation about PTSD and coping strategies was given in a group format. The treatment schedule is summarized in Table 2. Participants stayed at the hospital hotel at Södra Älvsborgs Sjukhus during the treatment, where all meals were served. Participants had the possibility to use telephone coaching outside sessions during the treatment. Therapist rotation was used for both PE and EMDR, with the assumption that less focus on the relationship between patient and therapist would facilitate increased focus on processing traumatic memories (33). Typically, participants met 8 different therapists during the treatment. The therapists were all trained in PE or EMDR, or both methods. Information on treatment progress was reported and discussed between therapists at a 30-min meeting every morning.

**Table 2:** Treatment schedule.

Morning
Physical activity (PA) 30 min
Prolonged Exposure 90 min
PA 30 min
Lunch

Afternoon
PA 60 min
EMDR 90 min
PA 30 min
Dinner

Evening
Psychoeducation^a^

a Except day 4 och 8.

### 2.6. Data analysis

All statistical analyzes were performed with IBM SPSS for Windows (version 28). The authors were blinded to the identity of the participants during data management procedures. Data were screened for data-entry errors and outliers, and assumptions associated with planned statistical analysis were checked *via* explore function in SPSS. Means, standard deviation and frequency for demographic and clinical variables in the sample were calculated. The total missing data was 6.5%. No systematic pattern could be found in the missing data. Imputation was used for missing data, the value for the previous measure was repeated. To determine whether the intervention brought a change in PTSD symptoms (measured by PCL-5) and DSO symptoms (measured by ITQ-part 2) from pre-to posttreatment and 3-month follow-up, a repeated measures analysis of variance (ANOVA) was used, two-tailed using a 95% level of significance. Two effect size measures were calculated: partial eta squared and Cohens’*d* for paired samples. To determine to which degree the intervention led to a clinically meaningful change at posttreatment and 3-month follow-up on primary outcome measure (PCL-5), two definitions were used. National Center for PTSD (NCPTSD) (34) recommend 10 points as minimum change. Marx et al. (22) recommend a PCL-5 value of <28 points at posttreatment to measure of clinically meaningful treatment effect. The proportion of participants reaching thresholds at pre-, posttreatment and 3-month follow-up was calculated. The total value of SSI items 4 and 5 during the interview was used to determine whether suicidal intentions changed during the intervention. Friedman’s test was used to compare median values pretreatment, midtreatment, posttreatment and 3-month follow-up.

## 3. Results

### 3.1. Sample characteristics and protocol deviations

Of 11 participants, 83% were female and 17% male. The mean age was 26,2 (sd 7,73). 72% were living with their spouse or primary family, 28% in a single household. 36% were employed, and 18% had a temporary employment. Mean year of education was 11,45 (sd 1,82). 27% of the participants met the criteria for C-PTSD diagnosis. Sexual violence was the most common traumatic event (92%), followed by physical violence (82%). For both sexual and physical violence, the traumatic event was experienced before age 13 in 45% of the cases. 45% had a history of previously unsuccessful trauma-focused treatment. Nine participants completed the whole treatment. Two participants ended treatment earlier than expected due to reduced PTSD symptoms and fully processed trauma memories, one participant on day 2 and one participant on day 3. Both participants completed posttreatment evaluation and were included in the analysis.

### 3.2. Treatment results

The mean and standard deviation for PTSD symptoms (PCL-5) and DSO symptoms (ITQ-part 2) are presented in Table 3. The ANOVA of PTSD symptoms indicated a significant change over time (Wilks Lambda = 0.18, *F* (df1 = 2, df2 = 9) = 19.96, *p < 0*.001, partial eta squared = 0.82). The ANOVA of DSO symptoms (Table 3) indicated a significant change over time (Wilks Lambda = 0.44, *F* (df1 = 2, df2 = 9) = 5,75, *p* < 0.025, partial eta squared = 0.56). Effect sizes comparing pretreatment to posttreatment and 3-month follow-up respectively, were large for both PTSD and DSO symptoms. 91% of participants who completed treatment reached the threshold for clinically meaningful change in PTSD symptoms at posttreatment and 100% at 3-month follow-up. Using alternative definitions of clinically meaningful change in PTSD symptoms recommended by Marx et al. (22), 72% reached the threshold at posttreatment and 3-month follow-up. At posttreatment, 91% of participants completing treatment no longer meet the criteria for PTSD according to ICD-11. No participants met the criteria for C-PTSD. At 3-month follow-up, none of the participants met the criteria for PTSD.

**Table 3:** PCL-5 and ITQ part 2 at pre-, posttreatment and 3-month follow-up (*n* = 11).

	Pretreatment		Posttreatment			3 months				
	Mean	SD	Mean	SD	*d*	Mean	SD	*d*	*ɳ^2^*	*p*
PCL-5	56.64	10.34	22.73	14.89	1.69	20.09	12.38	1.92	0.816	<0.001
ITQ-DSO	15.09	4.72	8.9	6.09	0.98	7.5	5.79	1.00	0.561	<0.025

d = Cohens ‘d, ɳ^2^ = partial eta squared, p = level of significance.

### 3.3. Treatment process

**Figure 1:**
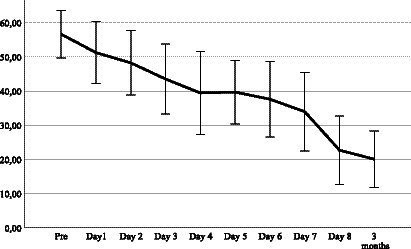
Mean and standard deviation PCL-5 per treatment day and follow-up (*n* = 11).

In the daily PCL-5 scores (Figure 1), no symptom exacerbation could be observed at group level. At group level, no part of the treatment process could be associated with worsening or breach of a positive trend in PCL-5 scores throughout the treatment.

### 3.4. Potential risks

One of the participants (9%) who completed treatment (*n* = 11) had an increased score (+10 points) on PCL-5 at posttreatment compared to pretreatment. The same participant, however, had a lowered score on PCL-5 at 3-month follow-up and no longer met the criteria for PTSD. Regarding change in suicidal intentions during treatment, Friedmans test (0.05 significance level) indicated no statistically significant difference between any of the measuring points (pretreatment, day 4, posttreatment, 3-month follow-up) (*x*^2^ (df = 3, n = 11) = 6,000, *p = 0.112*). One participant used the coaching telephone on two occasions outside office hours to manage anxiety, this participant completed treatment.

## 4. Discussion

The primary purpose was to explore whether an 8-day ITP is feasible and safe for patients with severe PTSD when applied in Swedish public healthcare. The results showed that a reduction in PTSD symptoms could be observed at posttreatment and at 3-month follow-up, with large effect sizes. A positive trend was observed at group level throughout the treatment, with no indication of a symptom exacerbation. DSO symptoms were also reduced during the treatment. The results suggest that the 8-day program could be a useful treatment option even if the target population suffers from previously treatment-resistant PTSD or psychiatric comorbidity. These results align with previous studies showing that the 8-day ITP is an effective treatment for not only severe PTSD but also C-PTSD (11, 13). Like previous studies (15) the positive results suggest that the 8-day ITP is a robust treatment that can be applied in various settings. At an individual level, the results were positive as a majority of the participants had a clinically meaningful change in symptom severity and no longer met the criteria for PTSD posttreatment. However, the results presented here should be interpreted cautiously due to the small sample size.

The results were also positive from a safety and risk perspective. The number of dropouts (8%) in this study seems acceptable compared to the average number of dropouts in PTSD treatment (18%) given week by week (35). This finding also aligns with studies showing lower rates of treatment abortion in intensive PTSD treatments compared to traditionally paced treatments (9, 36). One person aborted treatment due to escalating risk in home environment. Although reasons for aborting were unrelated to the treatment itself, this case highlights the importance of assessing current stressors before treatment. One person in this study reported an increase in PTSD symptoms posttreatment but recovered at 3-month follow-up. We suggest that potentially inhibitive factors in the treatment format and the participants should be further explored. Yet another important risk that should be continuously monitored is potential increase in suicidal intentions. Previous studies of the 8-day intensive treatment have not shown an increased risk of suicidal behaviors, even in individuals with C-PTSD (13). In this pilot, we could also further explore the participants’ attitudes and intentions toward suicidal behavior. No degree of worsening could be observed in either reported suicidal ideation or adverse events. Thus, no indication of increased risk or harmful effects due to the intensive format could be detected.

### 4.1. Limitations

Both due to design and small sample size, this feasibility study has several limitations regarding which conclusions can be drawn. The small sample reduces the possibility of generalizing the results to patients seeking healthcare due to severe PTSD. No control group was used, which make conclusions regarding the actual effect of the treatment impossible. Apart from studies with larger sample sizes with control group, there is also a need to examine the effect of the 8-day ITP in patients diagnosed with C-PTSD in Swedish public healthcare, as only three patients diagnosed with C-PTSD were included in this study.

### 4.2. Conclusion and recommendations

The results indicate that an 8-day ITP could be a useful treatment for severe PTSD in a Swedish public healthcare setting. A positive change in symptom levels was observed and no adverse events or increase in suicidal ideation was detecded. Based on these results, we conclude that support for the 8-day ITP as a safe form of treatment has grown even further. Therefore we believe this form of treatment should be further explored as an option to traditionally paced treatments in public healthcare, with larger sample size and further exploration of how the presence of C-PTSD interacts with treatment effects. Although the short time in which substantial symptom relief can be achieved is appealing in itself, there is also a need for further exploration of potential advantages or disadvantages of the 8-day ITP by comparing treatment effects directly to traditionally paced PTSD treatments.

## Data availability statement

The datasets presented in this article are not readily available because the ethics approval of the current project does not allow sharing of data. On request, the possibility of sharing data in accordance with the ethics approval will be reviewed. Requests to access the datasets should be directed to hannes.gahnfelt@vgregion.se.

## Ethics statement

The studies involving human participants were reviewed and approved by Etikprövningsmyndigheten i Sverige (Ethics Commitee in Sweden). The participants provided their written informed consent to participate in this study.

## Author contributions

HG, PC, and CB equally contributed to the conception and design of the project. CB was the project leader. HG performed the statistical analysis and wrote the first draft of the manuscript. PC and CB wrote sections of the manuscript. All authors contributed to the article and approved the submitted version.

## Funding

This work was supported by Forsknings och utvecklingsenheten (Research and development unit) Södra Älvsborg, Grant/Award: VGFOUSA-963589. Apart from this grant, research has been performed within the authors’ clinical and daily practice at Södra Älvsborgs Hospital.

## Conflict of interest

The authors declare that the research was conducted without any commercial or financial relationships that could be construed as a potential conflict of interest.

## Publisher’s note

All claims expressed in this article are solely those of the authors and do not necessarily represent those of their affiliated organizations, or those of the publisher, the editors and the reviewers. Any product that may be evaluated in this article, or claim that may be made by its manufacturer, is not guaranteed or endorsed by the publisher.
